# Bevacizumab for the treatment of metastatic colorectal cancer complicated by abdominal aortic aneurysm and mural thrombus: a case report

**DOI:** 10.3389/fonc.2024.1416349

**Published:** 2024-11-18

**Authors:** Yujun Li, Tong Wu, Muhammad Muddasar Saeed, Xiaonan Cui

**Affiliations:** Department of Oncology, The First Affiliated Hospital of Dalian Medical University, Dalian, China

**Keywords:** abdominal aortic aneurysm, bevacizumab, colorectal cancer, molecular targeting treatment, case report

## Abstract

Bevacizumab, a humanized monoclonal antibody targeting vascular endothelial growth factor (VEGF), was the first anti-angiogenic agent incorporated into metastatic colorectal cancer treatment strategies and demonstrated broad-spectrum anti-tumor efficacy. Commonly reported adverse events include hypertension, proteinuria, gastrointestinal perforation, bleeding, and thromboembolism. However, there are only a few reports on abdominal aortic aneurysms (AAA) as a complication of bevacizumab therapy. Given the high risk of fatal rupture with AAA, we present a case of bevacizumab-associated AAA to raise clinician awareness of this possible, rare, and serious adverse reaction.

## Introduction

In 2022, the International Agency for Research on Cancer reported that colorectal cancer ranks as the third most common cancer globally (9.6%) and the second leading cause of cancer-related mortality (9.3%). More than 50% of colorectal cancer patients will develop distant metastasis at various stages of the disease (1). Although colorectal cancer remains incurable, advancements in treatment have significantly improved outcomes for patients with metastatic colorectal cancer over the past two decades (2).

The progression of colon cancer is closely related to various biomolecules involved in tumor angiogenesis. The tumor vasculature plays a critical role in the progression of colon cancer by supplying oxygen and nutrients (3). Bevacizumab, a humanized monoclonal antibody targeting vascular endothelial growth factor (VEGF), was the first anti-angiogenic agent included in the therapeutic strategy for metastatic colorectal cancer. By binding specifically to VEGF, bevacizumab inhibits tumor neovascularization and blocks the VEGF receptor, thereby blocking the angiogenesis signaling pathway (4). Additionally, bevacizumab has been successfully combined with multiple chemotherapeutic agents for the treatment of recurrent or metastatic colon cancer.

The AVF2107g and E3200 trials have confirmed that adding bevacizumab to 5-Fu-based chemotherapy significantly improves progression-free survival (PFS) and overall survival (OS) in patients with metastatic colorectal cancer. Moreover, the TRC-0301 and ML18147 trials demonstrated the feasibility of continued bevacizumab treatment in patients with post-progression colorectal cancer. While bevacizumab demonstrated promising broad-spectrum anti-tumor activity, its cardiovascular adverse effects warrant careful attention. Given the life-threatening risk associated with abdominal aortic aneurysms (AAA) rupture, we present a case of AAA potentially induced by bevacizumab to raise clinician awareness of this rare but serious adverse effect.

## Case presentation

A 65-year-old male with no history of hyperlipidemia, hypertension, and cardiovascular disease was admitted to the hospital in August 2022 complaining of abdominal distension and a change in stool pattern for three months. Abdominal contrast-enhanced computed tomography (CT) showed a rectal mass and multiple low-density lesions in the liver, and pathological analysis of the tumor revealed adenocarcinoma. Laparoscopic radical rectal resection was performed, and the genetic testing showed wild-type NRAS, KRAS, and BRAF. The final diagnosis was postoperative rectal cancer pT4aN1bM1, stage IVA, with multiple liver metastases.

The patient initially underwent six cycles of the first-line XELOX plus cetuximab regimen, followed by maintenance therapy with cetuximab until the twentieth cycle. However, subsequent reexamination found that the liver metastases had progressed compared to before. According to the latest CSCO and NCCN guidelines, the second-line mXELIRI regimen was administered every 21 days for a total of 6 cycles, involving irinotecan (200 mg/m^2^) and capecitabine (800 mg/m^2^), in combination with 500 mg of bevacizumab (7.5 mg/kg), supplemented by relevant protective and supportive treatments. During this period, periodic reexamination CT scans showed that the lesions remained stable, while the patient’s blood pressure fluctuated between 96–136/90–96 mmHg during treatment. Due to the history of diabetes, blood glucose levels were controlled between 6.27–11.39 mmol/L by taking hypoglycemic drugs regularly. Unfortunately, the patient refused further treatment.

**Figure 1:**
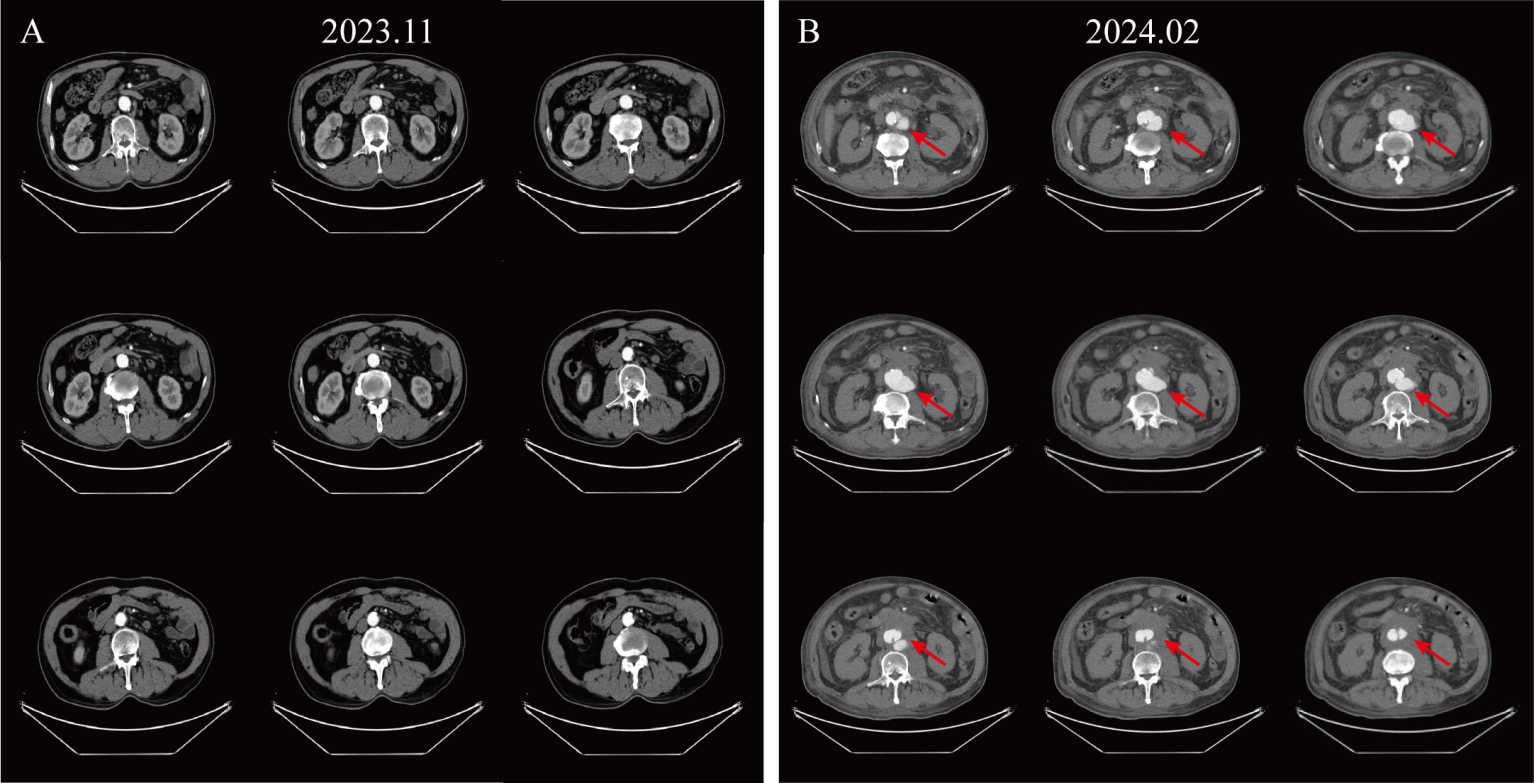
Enhanced CT images illustrating disease progression. **(A)** Enhanced CT of the abdomen at the patient’s last chemotherapy. **(B)** Enhanced CT presents a newly emerging localized aneurysmal dilatation of the abdominal aorta, considered an intramural hematoma with ulceration (Crawford type IV), measuring about 7.6 cm in diameter at its widest point.

After two months of discontinuing treatment, the patient was re-admitted to the hospital due to sudden severe abdominal pain, with a blood pressure of 140/96 mmHg. Enhanced CT revealed a newly formed local intramural ulcer of the AAA with intramural hematoma (**Figure 1**). The risk of AAA rupture was high, but the patient refused immediate surgery. We recommend that the patient continue to monitor blood pressure, take anticoagulant medication, and undergo surgical treatment as soon as possible.

Based on the patient’s prior history of anti-tumor drug treatment history, pre- and post-imaging characteristics, and related pharmacological effects, we considered that this is a severe adverse event of AAA triggered by bevacizumab treatment.

## Discussion

An abdominal aortic aneurysm is characterized by a localized dilation of the abdominal aorta, defined as an increase of 50% or more above the normal arterial diameter. Clinically, an AAA diagnosis is made when the aorta diameter exceeds 30 mm (5). Symptomatic AAA, which commonly presents with pain or rupture, often necessitates surgical treatment. Notably, the mortality rate associated with ruptured AAA exceeds 90% (6).

Previous studies indicate that the VEGF signaling pathway is associated with activating the phosphatidylinositol-3-kinase (PI3K)-AKT signaling pathway during angiogenesis. Inhibiting this signaling pathway may lead to matrix metalloproteinase 9 (MMP-9) overexpression, resulting in extracellular matrix degradation (7). Additionally, blocking VEGF disrupts the repair process of damaged vascular endothelium, impairs the regenerative ability of endothelial cells, and ultimately contributes to tissue exposure and endothelial cell apoptosis (8). However, there are still relatively few studies exploring the relationship between anti-angiogenic drugs and aneurysms. A pharmacovigilance study conducted in Japan, using data from the VigiBase database, included worldwide cases of arterial entrapment and aneurysms associated with anti-angiogenic drugs, demonstrating an association between anti-angiogenic drugs and the development of aneurysms through disproportionality analysis. These findings suggest that anti-angiogenic therapies may cause damage to the arterial wall (9).

From 2008 to 2024, we searched PubMed using the search terms “bevacizumab” and “aortic aneurysm” and we found four relevant case reports (10–13). These patients included three men and one woman, with a median age of 66.5 years (range: 54–74 years). Three patients had a history of hypertension, a recognized risk factor for abdominal aortic aneurysm rupture (14). Bevacizumab can reduce the level of nitric oxide and its metabolites, then increase endothelin levels, which leads to elevated blood pressure, atherosclerotic plaque instability, and plaque rupture. However, our cases had no history of hypertension, and no transient increases in blood pressure were observed before or after anti-angiogenic drug treatment. This finding suggests that bevacizumab may damage the arterial wall, leading to the development of aneurysms as a secondary consequence of these injuries from a variety of causes. In addition, the AAA in our cases occurred after four cycles of bevacizumab treatment, aligning closely with the previously reported median onset time of six cycles (range: 3–28 cycles). Therefore, the potential risk of AAA formation needs to be considered in patients undergoing bevacizumab therapy, regardless of the duration of treatment.

In November 2022, the latest guidelines for the diagnosis and management of aortic disease recommended surgical repair for male patients with unruptured AAA measuring 5.5 cm or higher (15). Besides, previous studies have indicated that the rupture probability of AAA is positively correlated with the size of the aneurysm. For AAAs with an inner diameter of 7–7.9 cm, the annual rupture risk ranges from 20%–40% (16). In our case, the patient’s abdominal CT revealed a maximum aortic diameter of approximately 7.6 cm, prompting a recommendation for surgical intervention following a vascular surgery evaluation. Unfortunately, the patient ultimately gave up surgery for personal reasons and opted for discharge for follow-up observation.

In conclusion, while there is limited experience regarding bevacizumab-associated AAAs, their occurrence can significantly impact patient prognosis and even lead to death. Therefore, patients who have previously received or are currently undergoing anti-angiogenic targeted therapy, such as bevacizumab, should be concerned about the potential risk of developing AAAs. If bevacizumab is deemed the optimal standard regimen, careful management of the drug dosage is essential to ensure efficacy while minimizing associated adverse events. Furthermore, any sudden onset of abdominal pain should prompt immediate evaluation and treatment, with AAA as a potential consideration. For patients with a history of AAA, the risks associated with anti-angiogenic targeted therapy should be thoroughly evaluated and avoided whenever possible.

## Data Availability

The original contributions presented in the study are included in the article/supplementary material. Further inquiries can be directed to the corresponding author.

## References

[1] Dekker E Tanis PJ Vleugels JLA Kasi PM Wallace MB . Colorectal cancer. Lancet (London England). (2019) 394:1467–80. doi: 10.1016/s0140-6736(19)32319-0 31631858

[2] Riedesser JE Ebert MP Betge J . Precision medicine for metastatic colorectal cancer in clinical practice. Ther Adv Med Oncol. (2022) 14:17588359211072703. doi: 10.1177/17588359211072703 35237350 PMC8882813

[3] Zhou W Zeng T Chen J Tang X Yuan Y Hu D . Aberrant angiogenic signaling pathways: Accomplices in ovarian cancer progression and treatment. Cell Signalling. (2024) 120:111240. doi: 10.1016/j.cellsig.2024.111240 38823664

[4] Liu Z-L Chen H-H Zheng L-L Sun L-P Shi L . Angiogenic signaling pathways and anti-angiogenic therapy for cancer. Signal Transduction Targeted Ther. (2023) 8:198. doi: 10.1038/s41392-023-01460-1 PMC10175505 37169756

[5] Golledge J Thanigaimani S Powell JT Tsao PS . Pathogenesis and management of abdominal aortic aneurysm. Eur Heart J. (2023) 44:2682–97. doi: 10.1093/eurheartj/ehad386 PMC10393073 37387260

[6] Mulatti GC Joviliano EE Pereira AH Fioranelli A Pereira AA Brito-Queiroz A . Brazilian society for angiology and vascular surgery guidelines on abdominal aortic aneurysm. J Vasc Brasileiro. (2023) 22:e20230040. doi: 10.1590/1677-5449.202300402 PMC10648059 38021279

[7] Dai S Zhong Y Cui H Zhao J Li S . Aortic dissection induced by vascular endothelial growth factor inhibitors. Front Pharmacol. (2023) 14:1189910. doi: 10.3389/fphar.2023.1189910 37426822 PMC10327890

[8] Totzeck M Mincu RI Rassaf T . Cardiovascular adverse events in patients with cancer treated with bevacizumab: A meta-analysis of more than 20 000 patients. J Am Heart Assoc. (2017) 6:e006278. doi: 10.1161/JAHA.117.006278 28862931 PMC5586462

[9] Guyon J Gouverneur A Maumus-Robert S Bérard X Pariente A Bikfalvi A . Association between antiangiogenic drugs used for cancer treatment and artery dissections or aneurysms. JAMA Oncol. (2021) 7:775–8. doi: 10.1001/jamaoncol.2021.0210 PMC7974832 33734295

[10] Aragon-Ching JB Ning YM Dahut WL . Acute aortic dissection in a hypertensive patient with prostate cancer undergoing chemotherapy containing bevacizumab. Acta Oncol (Stockholm Sweden). (2008) 47:1600–1. doi: 10.1080/02841860801978905 PMC7217319 18607842

[11] Baek SU Kwon SI . Rupture of abdominal aortic aneurysm after intravitreal bevacizumab injection: A case report. J Med Case Rep. (2014) 8:48. doi: 10.1186/1752-1947-8-48 24520842 PMC3938302

[12] Dong W Sun Q Xu S Wu D Xu J Cheng L . Sudden aortic dissection: A cautionary tale for the unexplained back pain during bevacizumab treatment. Radiol Case Rep. (2023) 18:2366–9. doi: 10.1016/j.radcr.2023.03.055 PMC10172617 37179807

[13] Yajima K Koga A Okumura T Yamashita K Isogaki J Suzuki K . A patient with lung cancer experiencing abdominal aortic aneurysm rupture during bevacizumab treatment-case report. Gan to Kagaku Ryoho Cancer Chemother. (2019) 46:1449–51. 31530788

[14] O’Donnell TFX Landon BE Schermerhorn ML . The case for expanding abdominal aortic aneurysm screening. J Vasc Surg. (2020) 71:1809–12. doi: 10.1016/j.jvs.2019.10.024 31831309

[15] Isselbacher EM Preventza O Hamilton Black J 3rd Augoustides JG Beck AW Bolen MA . 2022 acc/aha guideline for the diagnosis and management of aortic disease: A report of the american heart association/american college of cardiology joint committee on clinical practice guidelines. Circulation. (2022) 146:e334–482. doi: 10.1161/cir.0000000000001106 PMC9876736 36322642

[16] Brewster DC Cronenwett JL Hallett JW Jr. Johnston KW Krupski WC Matsumura JS . Guidelines for the treatment of abdominal aortic aneurysms. Report of a subcommittee of the joint council of the american association for vascular surgery and society for vascular surgery. J Vasc Surg. (2003) 37:1106–17. doi: 10.1067/mva.2003.363 12756363

